# Genome-wide analysis of HIF-2α chromatin binding sites under normoxia in human bronchial epithelial cells (BEAS-2B) suggests its diverse functions

**DOI:** 10.1038/srep29311

**Published:** 2016-07-04

**Authors:** Meng-Chang Lee, Hsin-Ju Huang, Tzu-Hao Chang, Hsieh-Chou Huang, Shen-Yuan Hsieh, Yi-Siou Chen, Wei-Yuan Chou, Chiao-Hsi Chiang, Ching-Huang Lai, Chia-Yang Shiau

**Affiliations:** 1 Graduate Institute of Medical Sciences, National Defense Medical Center, Taipei, 11490 Taiwan; 2 Department of Anesthesiology, Cheng Hsin General Hospital, Taipei, Taiwan; 3 Graduate Institute of Biomedical Informatics, Taipei Medical University, Taipei, 11031 Taiwan; 4 Department of Pharmacology, National Defense Medical Center, Taipei, 11490 Taiwan; 5 Department and Graduate Institute of Biochemistry, National Defense Medical Center, Taipei, 11490 Taiwan; 6 School of Pharmacy, National Defense Medical Center, Taipei, 11490 Taiwan; 7 School of Public Health, National Defense Medical Center, Taipei, 11490 Taiwan

## Abstract

Constitutive functional HIF-2α was recently identified in cancer and stem cell lines under normoxia. In this study, BEAS-2B, a bronchial epithelial cell line, was shown to constitutively express active HIF-2α under normoxia and exhibit markers of pluripotency including Oct-4, Nanog, and sphere formation. Oct-4 expression was reduced after knockdown of HIF-2α under normoxia. Global enrichment analysis of HIF-2α demonstrated the diverse functions of HIF-2α under normoxia. Bioinformatics analysis of the enriched loci revealed an enhancer role of HIF-2α binding sites, involvement of HIF-2α interacting proteins, and enriched *de novo* motifs which suggest the diverse role of HIF-2α in pseudohypoxia. The low ratio of the discovered loci overlapping with those revealed in cancer cell lines 786-O (16.1%) and MCF-7 (15.9%) under hypoxia indicated a prevailing non-canonical mechanism. Hypoxia had positive, marginal or adverse effects on the enrichment of the selected loci in ChIP-PCR assays. Deletion of the N-terminal activation domain (N-TAD) of HIF-2α disrupted the reporting activity of two of the loci annotated to ELN and ANKRD31. Hypoxia incurring abundance variation of HIF-2α may misrepresent the N-TAD functions as canonical hypoxia inducible features via C-TAD activation. Elucidation of the pseudohypoxia functions of constitutive HIF-2α is useful for resolving its role in malignancy and pluripotency.

The cellular response to limited oxygen tensions is primarily via induction of the hypoxia inducible factors, HIF-1α and HIF-2α which are involved at crucial stages of embryonic development^1^,^2^,^3^, homeostasis^4^,^5^, and cancer pathogenesis^6^,^7^ such as sensing nutrient availability and intracellular pH, regulation of anaerobic glycolysis, modulation of the tumor microenvironment, and cell survival. HIFα protein molecules tend to become hydroxylated under normoxia at an oxygen dependent degradation domain by prolyl hydroxylase (PHD) leading to their ubiquitination and subsequent degradation via the von Hippel-Lindau protein (VHL)-proteasomal route^8^,^9^. Asparaginyl hydroxylase (also named factor inhibiting HIF, FIH) hydroxylates the C-terminal activation domain (C-TAD) leading to diminished recruitment of co-activator CBP/P300 and hence much reduced activation function^8^,^10^. Both PHD and FIH are members of the dioxygenase family which utilize molecular oxygen as a co-substrate for the hydroxylation of the HIFαs^11^,^12^. The former affects the abundance of HIFα and the latter exerts tight regulation on HIFα C-TAD activity with a higher affinity for oxygen than the former^13^. Thus, it is widely believed that hypoxia activated HIFs primarily through interaction between the C-TAD and CBP/P300^14^,^15^. To contrast with the results of the reported oxygen tension dependent inducible features, it is of considerable interest to investigate further some types of cells such as stem cells^4^, cancer stem cells^16^,^17^, and certain cancer cells^5^,^17^ that have been shown to exhibit constitutive expression of HIF-2α under normoxia. HIF-2αlikely has a major role in malignant transformation and maintenance of the pluripotent state^2^,^10^,^17^,^18^. HIF-2α molecules which have frequently been shown functional under normoxia though their C-TAD can still be inhibited by FIH^17^,^19^. Its underlying mechanism is very intriguing. Knockdown of PHD2 in HeLa cells intended to stabilize HIFs under normoxia was shown to partially induce a set of genes characterizing hypoxia, whereas combined silencing of both PHD2 and FIH induced a full set of genes characterizing hypoxia^20^. A conceivable mechanism for this is that interaction of HIF-2α with other transcription factors such as ETS family, NEMO, c-Myc and those cited in literature^12^,^21^,^22^,^23^ mutually enhances their transcription function, , indirectly resulting in activation of a set of genes under normoxia. Furthermore, it has been postulated that such induction of a partial set of genes under normoxia could be mediated by interaction of other domains, such as the N-TAD rather than the C-TAD, with distinct co-activators which were shown to be different from those of its C-TAD^9^,^24^,^25^. C-TAD truncated HIF-2α was able to activate both HIF enriched elements and artificial promoters^9^,^20^. The N-TAD of HIF-2α has major differences in sequence from that of HIF-1α^25^,^26^. “Domain-swapping” experiments between HIF-1α and HIF-2α provided clear evidence of gene selectivity which was attributed to the N-TAD of HIF-α^25^,^27^. The PAS domain was also reported to recruit specific co-activators and to contribute to the target specificity of HIF-2α^28^,^29^. Genes uniquely regulated by HIF-2α but not by HIF-1α include some implicated in malignant transformation, such as PAI-1, CXCR4, TGFα, Flk-1 and some in pluripotency, such as Oct-4, Sox2, Nanog, and cyclin D^12^,^18^,^22^. Whether epigenetic cues plus environmental factors can favor the expression of stemness-related HIF-2α downstream genes and/or suppress expression of malignancy-related HIF-2α downstream genes in a normal cell is of considerable interest and may reveal novel gene regulatory networks.

Given the considerable interest in the versatile biological functions of this kind of proteins, many gene profiles^30^,^31^,^32^ and chromatin enrichment analyses of HIFs^33^,^34^,^35^,^36^ have been performed under hypoxic conditions mostly in neoplastic cells. It is of interest to exploit the broad, hypoxia-inducible, and beneficial functions of HIFs in lung. The constitutive expression of HIF-2α but not HIF-1α was found in lung epithelial cells BEAS-2B to maintain viability and to exhibit a protective function against H_2_O_2_ insult (unpublished observation). Also, some markers and features characterizing pluripotency were found in BEAS-2B cells under normoxia. Furthermore, a genome wide analysis of HIF-2α chromatin enrichment was conducted in BEAS-2B cells in the absence of HIF-1α competition for enrichment at certain sites; this protocol contrasts with previous investigations under hypoxia conditions^33^,^34^,^35^,^36^, in which HIF-1α was shown to up-regulate histone demethylase and histone modification could dramatically affect chromatin structure and gene expression^37^,^38^. High enrichment of HIF-2α at loci was confirmed by using ChIP-PCR and reporter assays under normoxia. Enrichment of some of the candidate loci were shown less or un-responsive to hypoxia or sometimes even labile to hypoxia. Both N-TAD and C-TAD were found to be involved in specific enrichments with different weightings to each of the loci. In combination with bioinformatics analysis of sequence motifs, the present work provides insights into the pseudohypoxia functions of HIF-2α in BEAS-2B.

## Results

### Expression of pluripotency-related markers and a sphere culture of BEAS-2B under normoxia

**Figure 1:**
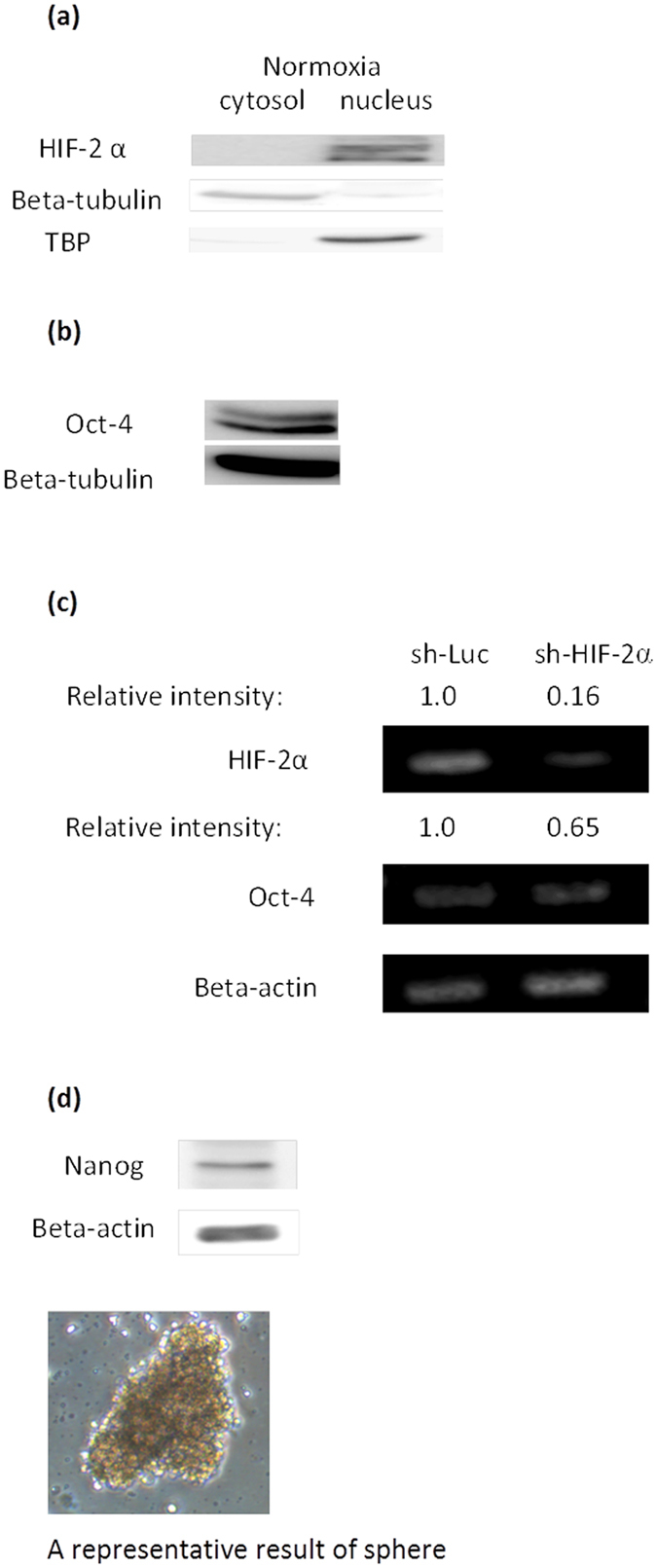
Expression of pluripotency related markers and a sphere culture of BEAS-2B under normoxia. (**a**) HIF-2α expression was detected in the nucleus of BEAS-2B under normoxia by western blotting. Beta tubulin and TATA binding protein (TBP) were used as protein markers for the cytosol and nucleus fraction, respectively. (**b**) Western blotting for Oct-4. (**c**) For knockdown of HIF-2α, BEAS-2B cells were transfected with shRNA targeting HIF-2α and sh-Luc was used as control. (**d**) Western blotting of Nanog and a culture of BEAS-2B containing floating spheres. Representative result from two or three experiments was presented.

In hypoxic but not normoxic embryonic stem cells, HIF-2α translocates into the nucleus to regulate pluripotency markers such as Oct-4, Sox2, and Nanog^39^,^40^. Generally, stem cells start to differentiate following the disappearance of Oct-4 expression. It is not common to detect Oct-4 expression in a differentiated normal cell. However, intriguingly, in our study, both constitutive expression of HIF-2α in the nucleus (Fig. 1a) and Oct-4 (Fig. 1b) were detected in BEAS-2B under normoxia while HIF-1α was not (unpublished observation). Knockdown of HIF-2α led to reduced Oct-4 expression (Fig. 1c) even under normoxia. In addition, expression of Nanog and a culture of BEAS-2B containing floating spheres (Fig. 1d) were also observed under normoxia. These results are similar to reports that HIF-2α expressed in several malignant and stem cells is functional under normoxia^8^,^17^,^41^. However, the possible pseudohypoxia function of HIF-2α has not been delineated. Therefore next, a genome wide analysis of HIF-2α under normoxia was conducted to explore its possible function and features.

### ChIP-Seq and analysis of NGS data

Further investigation of the enrichment of nuclear HIF-2α on chromatin in BEAS-2B under normoxia was performed by ChIP-Seq analysis. After alignment of our ChIP-Seq data to hg19, more than 10 million mapped reads enriched by HIF-2α antibody (10,313,454) and IgG (10,469,717) respectively, were identified (Supplementary Data 1 Table S2). CisGenome software v2.0 was adopted for further analysis of the mapped reads and we selected “Two Sample Peak Calling” to look for binding peaks from IP versus control samples (http://www.biostat.jhsph.edu/~hji/cisgenome/index.htm). The final criteria, as described in Methods, for analysis led to identification of 1,991 binding peaks (Supplementary Data 2) and the top 20 are listed in Table 1.

Among these binding peaks were loci corresponding to 1741 annotated closest genes, including 96 that were annotated as regulated areas for non-protein coding genes and several of them annotated to the same genes. ChIP-Seq analysis of HIF enrichments conducted for a few cancer cells under hypoxic condition has been reported by several groups^33^,^34^,^35^,^36^. These studies led to the discovery of more potential functional features of HIFs under hypoxia. A small portion of the annotated gene loci in this study coincided with those reported under hypoxic conditions, around 16.1% of those in MCF-7 which expresses both HIF-1α and HIF-2α under hypoxia and 15.9% of those in 786-O, which expresses only HIF-2α under normoxia but not HIF-1α even under hypoxia (Supplementary data 1 Table S3A,B)^34^,^36^. These overlapping annotated gene loci were previously attributed as hypoxia inducible features.

### Distribution of HIF-2α ChIP-Seq peaks

**Figure 2:**
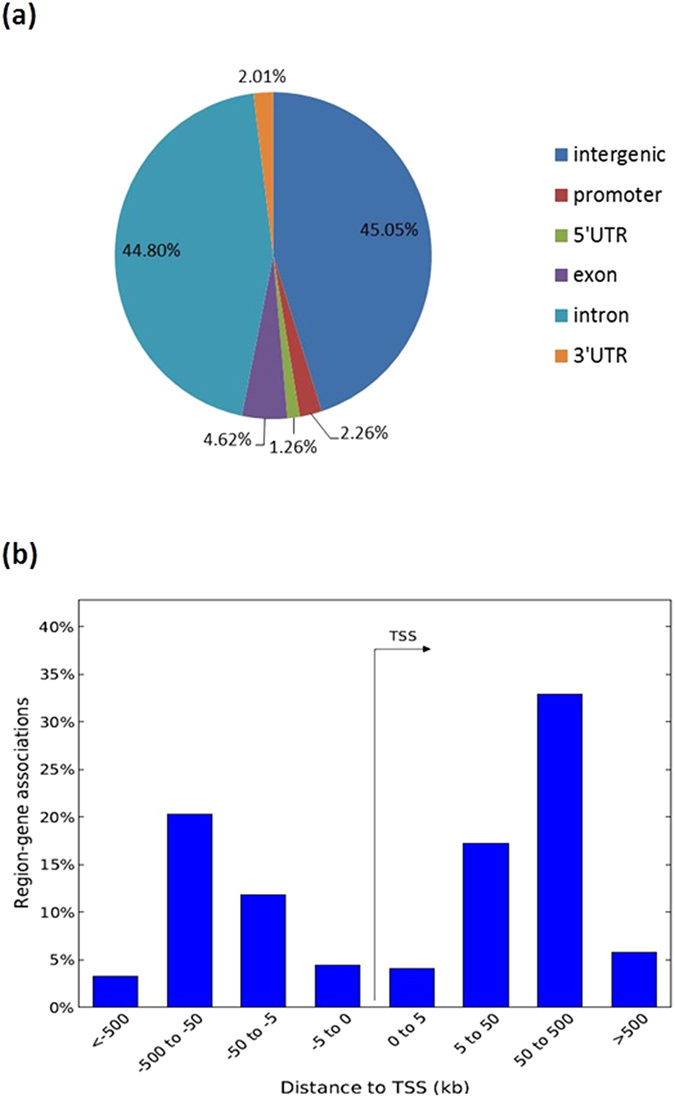
Distribution of HIF-2α ChIP-seq peaks in BEAS-2B under normoxia. (**a**) Pie chart illustrating the genomic locations of HIF-2α binding sites. (**b**) Bar graph showing the percentage of region–gene associations according to genomic regions’ distance to TSS computed by Genomic Regions Enrichment of Annotations Tool (GREAT) (http://bejerano.stanford.edu/great/public/html/).

Similar to the previously reported ChIP-Seq work on HIFs under hypoxia, it was revealed that HIF-2αenrichment occupied fewer promoters (2.26%) but more in introns (44.80%) and more in remote regions far away from the transcription start site (TSS) (Fig. 2a,b). HIF-2α enrichment is inclined to distal regions away from the TSS, implying a role for enhancers in its target regulation^34^,^36^,^42^. The actual proximal interactions between distant enriched loci and regulated genes may be brought about through three dimensional chromatin looping structure with the aid of other proteins and remains intriguing, as what was studied about the regulation of cyclin D in 786-O by HIF-2α using chromatin conformation capture^36^.

### Differential enrichment of HIF-2α under normoxia and hypoxia

**Figure 3:**
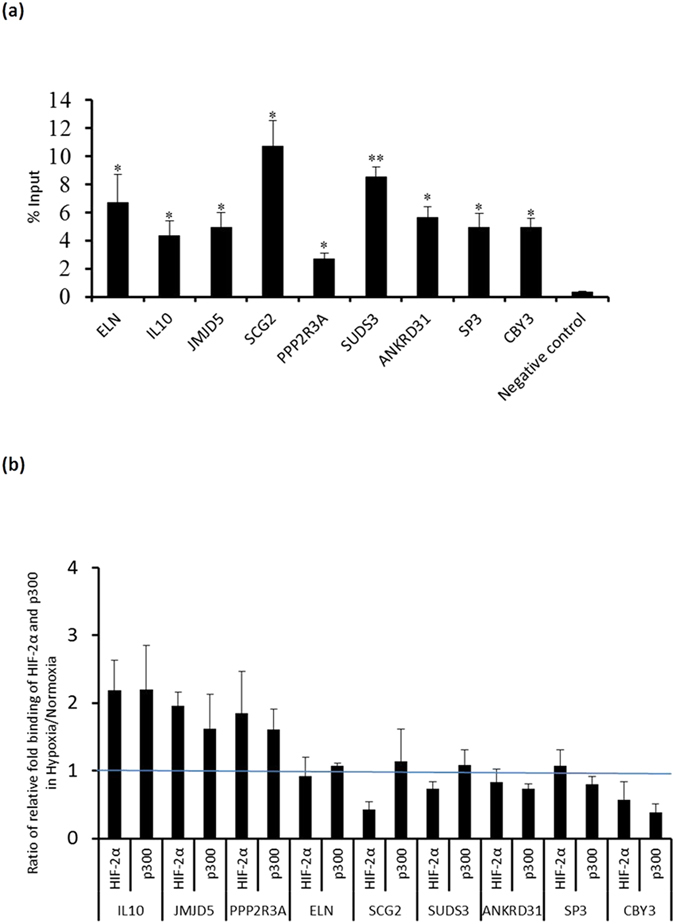
Chromatin enrichment of HIF-2α analyzed by using ChIP-PCR. (**a**) Representative enrichments of HIF-2α provided by ChIP-Seq analysis were further verified using semiquantitative PCR. Data are presented as signal relative to that of input DNA (100%). *P < 0.05 and **P < 0.01 vs. negative control. (**b**) The confirmed enrichments were further subjected to hypoxia mimetic treatment using CoCl_2_ (150 μM). Antibody against HIF-2α or P300 was used with the same primer pair. Signal of semiquantitative PCR relative to that of input DNA was used to calculate the relative fold binding between hypoxia and normoxia. All values were estimated by using image J (mean ± SD, n = 3).

**Figure 4:**
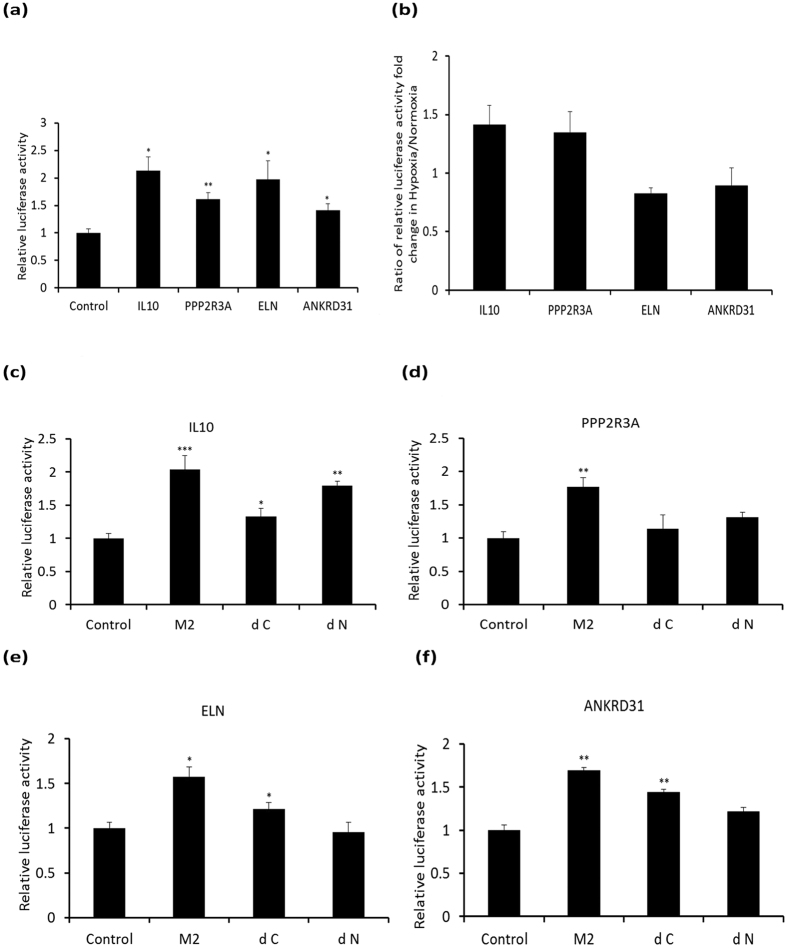
Reporter assay on enriched loci annotated to IL10, PPP2R3A, ELN and ANKRD31. Cypridina luc reporter plasmid loaded with or without sequences of enriched loci annotated to IL10, PPP2R3A, ELN or ANKRD31was used for the assay. Cypridina luciferase activity was normalized to the red firefly luciferase activity. (**a**) Luciferase activity is presented relative to empty vector in BEAS-2B under normoxia. (**b**) Relative luciferase activity between hypoxia (treated with 150 μM CoCl2) and normoxia. (**c**–**f**) Relative luciferase activity when the reporter plasmid was co-transfected with HIF-2α variants in HEK293 in a separate expression plasmid from Addgene. M2, a stabilized full length HIF-2α mutant, dN or dC, the N-TAD or the C-TAD truncated HIF-2α respectively. Data are shown as mean ± SD, n = 3. *P < 0.05; **P < 0.01 and ***P < 0.001 vs. control.

Among representative loci chosen for verification of enrichment by using ChIP-PCR assay, nine of them here were shown highly enriched with HIF-2α under normoxia with percentage input ranging from 2.71% to 10.73% (Fig. 3a). A fragment of β-actin exon was chosen as a negative control^34^. In comparison to other previously reported data for hypoxic conditions, enrichments of the selected loci under normoxia were relatively high^34^,^35^,^36^,^43^. Hypoxia was reported to activate HIF transcriptional activity via recruitment of the co-activator P300 to the C-TAD^14^,^15^. In the case of BEAS-2B, abundance of HIF-2α was not apparently affected under hypoxia in our study. Thus, the effect of hypoxia on enrichments of HIF-2α and P300 on the representative binding loci was further investigated. HIF-2α enrichments at those loci annotated to IL10, JMJD5, and PPP2R3A respectively were shown apparently enhanced under hypoxia mimetic condition using CoCl_2_ (Fig. 3b). A concomitant rise of P300 enrichment at these sites was also observed under hypoxia. These data reflect the notion as documented in the literature that cooperation between the N-TAD and C-TAD facilitates some of the HIF transcription activity under hypoxia^15^,^25^. On the other hand, hypoxia exerted relatively less effect on enrichment of HIF-2α at loci annotated to ELN, SP3, and ANKRD31. We surmised that the less responsiveness to hypoxia was because there was less involvement of the C-TAD of HIF-2α in enrichment. Less involvement of the C-TAD was also reflected in less responsiveness of the other P300 enrichments to hypoxia except that at the locus annotated to CBY3. The apparent adverse hypoxia effect resulting in diminished enrichment of HIF-2α at those loci annotated to CBY3 (with high error bar), SCG2, and SUDS3 likely implies unknown interactions between HIF-2α and chromatin possibly involving recruitment of repressors^4^. The obvious inhibitory effect on enrichments of both HIF-2α and P300 could enable gene down-regulation , an area of study that remains largely unexplored. Next, four of the representative loci were selected for luciferase reporter assay to determine their activation function(s) with HIF-2α (Fig. 4a). All of the four loci were shown to be active relative to the backbone vector. Further hypoxia treatment to the reporter assay on these four loci consistently showed a response pattern similar to that of enrichment (Fig. 4b). The loci annotated to IL10 and PPP2R3A, wherein enrichments were increased under hypoxia, were also shown to respond positively to hypoxia in the established reporter assay. The loci annotated to ELN and ANKRD31, at which enrichments were less responsive to hypoxia, resulted in a minor scale of variation of observed reporter activity with a reduction similar to that of HIF-2α but not P300 enrichments under hypoxia. Further reporter assays with N-TAD or C-TAD truncated HIF-2α (dN and dC respectively) with these four loci in HEK293 cells clearly illustrated the relative contribution of HIF-2α domains in their activation function (Fig. 4c–f). A stabilized full-length active HIF-2α (M2) mutant (see details in Materials and Methods) was included for comparison. For both loci annotated to IL10 and PPP2R3A, the dC mutant demonstrated significantly more reduction of activation activity relative to M2, indicating relatively higher involvement of the C-TAD. This was consistent with the observation of their positive response to hypoxia. In the case of the locus for PPP2R3A, truncation of either domain resulted in loss of most of the activation activity indicating that there is a high degree of cooperation required. For both loci annotated to ELN and ANKRD31, dN elicited only minimal or no reporter activity relative to M2, although apparent reduction of activity was also seen for dC mutants. Deletion of the N-TAD disrupted most of the activation activity of HIF-2α in these loci suggesting an absolute requirement for the N-TAD for activation activity. Overall, both the N-TAD and the C-TAD and other domains of HIF-2α seem to be required to cooperate to achieve the observed activation activities of HIF-2α with different weightings at each representative locus selected for investigation.

None of the promoters of pluripotency-related genes which were reported to be bound by HIF-2α in stem cells, such as those of Oct-4, Sox2, and Nanog were found in our ChIP-seq data. ChIP-PCR assay did not show significant enrichment of HIF-2α at the reported Oct-4 promoter under normoxia or hypoxia (data not shown). Other factors for suppressing enrichment of the loci known to be bound by HIF-2α in stem cells could exist. However, further treatment with trichostatin A, a HDAC inhibitor, increased Oct-4 transcript and protein level (Supplementary Data 1 Figure S1 A,B) in BEAS-2B under normoxia, suggesting the existence of epigenetic modification in the regulation of stemness. It is also plausible that there are other as yet unrevealed cells and context dependent loci bound by HIF-2α for its expression.

### Analysis of HIF-2α binding motifs

**Figure 5:**
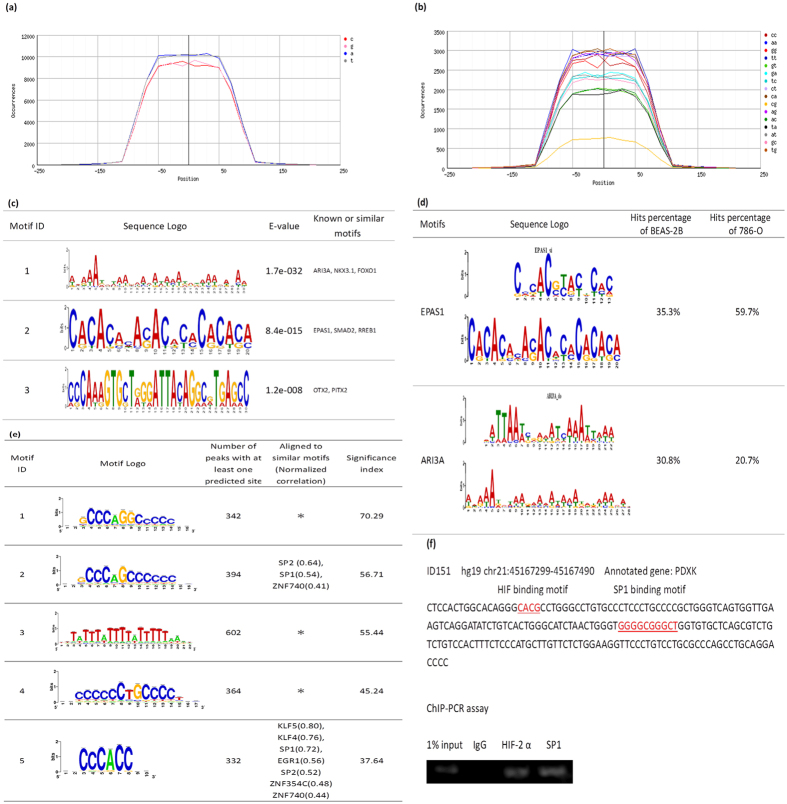
Analysis of HIF-2α binding motifs. HIF-2α binding profile of single nucleotide (**a**) or dinucleotide (**b**) composition generated by using RSAT. (**c**) The three most significant HIF-2α binding motifs under normoxia produced by MEME. (**d**) PWM Tools was used to scan the HOCOMOCO for EPAS1 motif 1 and ARI3A motif 2 against BEAS-2B and 786-O. (**e**) Analysis of HIF-2α potential heterodimers binding motif under normoxia. Results were generated by using dyad-analysis in RSAT. Significance index (sig) was computed by “sig = −log10(E value)” and E value = P value × number of tested character. Higher value means less error. *, *de novo* binding motifs that did not match those of any of known DNA binding proteins in the databank. (**f**) Co-enrichment of HIF-2α and SP-1 on Peak ID 151 annotated to PDXK.

Using RSAT for nucleotide analysis, it was apparent that occurrence of single nucleotide C or G were somewhat less than A or T by around 2% in a single nucleotide composition profile of −100 to 100 bps across the HIF-2α binding peaks (Fig. 5a). In the dinucleotide composition profile, CG was clearly much less than all other combinations within the HIF-2α binding peaks whereas CA and CT dinucleotide compositions were the most frequent (Fig. 5b). When MEME-ChIP^44^ and MEME^45^ using a longer oligomer up to 30 mers was applied to motif analysis, three significant motifs (E-value ≤ 0.05) were discovered (Fig. 5c). There were 133, 13 and 6 sequences containing motif 1, motif 2 and motif 3, respectively (Supplementary Data 3). The remainder of the various peaks could have been derived from complex indirect interactions with other proteins as described below. Since the ChIP-Seq data of BEAS-2B was derived from experiments performed in the absence of HIF-1α under normoxia, the observed enrichment pattern of HIF-2α has been determined without any interference from competition of HIF-1α activities which have been previously reported to overlap with that of HIF-2α to a significant extent. Furthermore, HIF-1α is known to enable transcription of histone demethylase known to affect chromatin structure and hence gene expression^37^,^38^. It can be concluded that there were a small portion of genes commonly annotated between normoxic BEAS-2B and hypoxic MCF-7 based on HIF-2α enrichment and a significantly higher portion between normoxic BEAS-2B and hypoxic 786-O cells (Supplementary Data 1 Table S3A,B). Cell lineage defining factors could have also played a pivotal role in exerting cell-type specific effects. The renal cancer cell line 786-O fails to express HIF-1α under hypoxia but constitutively expresses HIF-2α even under normoxia. The 611 binding peaks of HIF-2α and its closest annotated genes were obtained by utilizing CisGenome 2.0 for re-analysis of the deposited ChIP-Seq data of 786-O (GSE34871) (Supplementary Data 2). The above three motifs of BEAS-2B and the 611 peaks of 786-O were matched using MAST^46^. There were approximately 4.4%, 9.2% and 3.6% of 611 sequences of 786-O ChIP-Seq containing motif 1, motif 2 and motif 3 with an *E*-value less than 10 and a position P-value of less than 0.0001, respectively (Supplementary Data 1 Figure S2A–C). As shown in Fig. 5d, motif 2 and motif 1 possess a similar pattern to the EPAS1 (HIF-2α) and ARI3A binding region in the databank, respectively. PWM Tools^47^ were applied to scan the HOCOMOCO^48^ with the motif of EPAS1 and the motif of ARI3A against BEAS-2B and 786-O (Fig. 5d). Results showed that the EPAS1 binding region was more frequent in 786-O (59.7%) than in BEAS-2B (35.3%) likely due to the fact that the experiment described with 786-O was performed under hypoxia which induced the complete activation of HIF-2α. Additionally, binding regions of ARI3A, which encodes a member of the AT-rich interaction domain family of DNA binding proteins and has clear roles in transcriptional regulation and chromatin structure modification, is more abundant in BEAS-2B (30.8%) than in 786-O (20.7%). These results imply that at least some common regulatory mechanisms exist between the hypoxia and normoxia response reactions.

In BEAS-2B, a low portion of peaks, i.e., 11.9% comprising the core hypoxia response element (HRE) RCGTG and a low ratio of overlapping content with loci presented via the strategy of hypoxia treatment of 786-O and MCF-7 indicated a prevailing non-canonical mechanism under normoxia^43^,^49^. For comparison, the reported canonical HRE enrichment in primary macrophage under hypoxia was 43%^43^. Hypoxia could have resulted in epigenetic modifications since activity of chromatin histone demethylases is oxygen dependent and some of them are known to be regulated by HIF-1α. Enrichment at loci lacking HRE may represent events where HIF-2α was tethered with other transcription factors (or via proteins indirectly) to enhance the specificity of one other, especially at loci far from TSS. It has been demonstrated that HIF-2α interacts with various protein partners, like ETS, NEMO, and MYC, resulting in mutual enhancement of transcription activities at various targets. A *de novo* search for HIF-2α heterodimer binding motifs was conducted using dyad-analysis of RSAT. Five of the predicted motifs are listed (Fig. 5e). Two of them were annotated to similar binding motifs of several proteins. However, three of them did not match any motif of known DNA binding proteins in the databank. They may be *de novo* and in fact elicit functions via long range chromatin interaction, which would merit further study. Enriched loci within binding motifs as above could be directly derived from enrichment of HIF-2α or indirectly via its interacting protein partners. The motif analysis provides important clues about the *de novo* influence of other protein factors to HIF-2α binding to each site of chromatin or the *de novo* influence of HIF-2α to binding of other proteins to other sites of chromatin and thus their site specific transactivation activity. For exemplification, we detected the co-enrichment of HIF-2α and SP1 on the locus represented by peak ID151 (Fig. 5f).

### Gene ontology (GO) and pathway enrichment analysis of annotated genes

**Figure 6:**
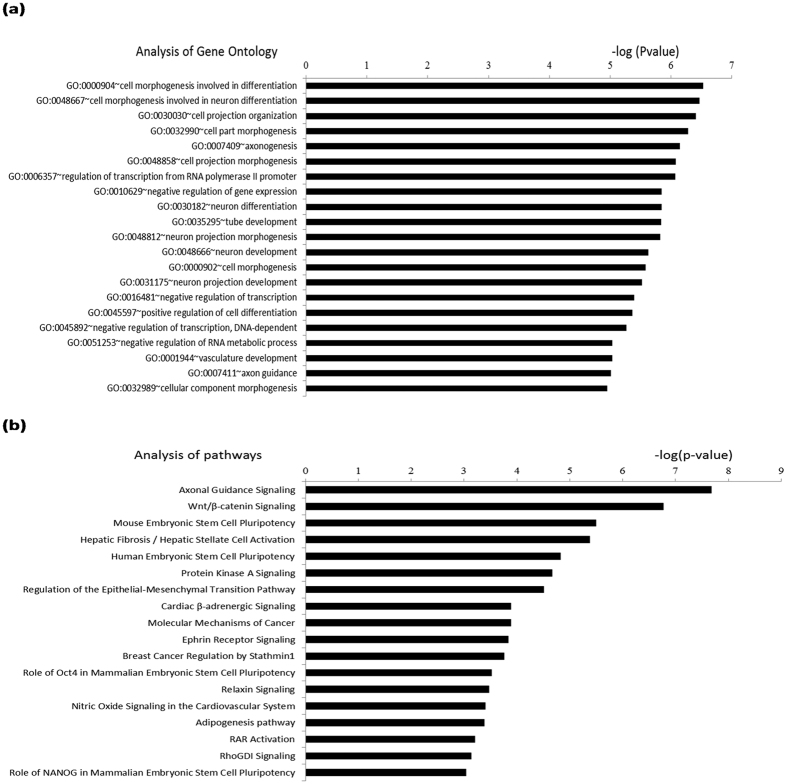
Analysis of GO and Pathway for HIF-2α annotated genes in BEAS-2B. (**a**) Analysis of GO was achieved by using DAVID, in which we adopted Gene Ontology/GOTERM_BP_FAT. Fold Enrichment >1.5 and P-Value, Bonferroni and FDR <0.05 were set as criteria for analysis. (**b**) Analysis of pathway by IPA and top 20 are shown according to the value of −log (P value).

Several HIF-2α downstream genes have been reported to be pluripotency-related and actively transcribed in stem cells and some have been successfully used for induction of pluripotent stem cells. For instance in this study, Oct-4 protein was detected though we could not find enrichment of HIF-2α at a locus in the promoter reported to be actively involved in transcription of Oct-4 in embryonic stem cells. It is not impossible that an unknown cell dependent locus exists to fulfill an enhancer role via long range interaction of chromatin. For comparison, a pathway analysis on published ChIP-Seq data indicated implication of HIF-2α in pluripotency in hypoxic MCF-7 but not in hypoxic 786-O cells^34^,^36^. Thus, GO analysis was performed on the 1741 annotated genes. HIF-2α was found to be associated with differentiation, regulation of transcription, vasculature development, and so forth (Fig. 6a). Interestingly, the truncated N-TAD of HIF-2α was also reported to debase the transcription activation of vascular endothelial growth factor (VEGF), a critical factor in vasculature development, implicating HIF-2α involvement with vasculature development via N-TAD^50^. Our results echo the concept that HIF-2α drives vascular development under less hypoxia as proposed by Koh and Powis, possibly suggesting less involvement with C-TAD of HIF-2α^50^,^51^. Using Ingenuity Pathway Analysis, HIF-2α was implicated in pluripotency of embryonic stem cells involving Oct-4 and Nanog (Fig. 6b)^34^. It was also shown that HIF-2α is involved in specific cancer-related pathways such as the Wnt signaling pathway. Several reports have actually linked HIF-2α to Wnt-β-catenin pathway in cancer cells^49^,^52^,^53^. Notably, constitutive expression of HIF-2α was also detected in colon cancer cells under normoxia and it was reported to be involved in Wnt signaling under normoxia^53^. Furthermore, HIF-2α brought more contribution to Wnt signaling under hypoxia, suggesting N-TAD and C-TAD of HIF-2α synergetic involvement in Wnt signaling but with different weightings^53^. Furthermore, in analysis of “Diseases and Functions” by IPA (Supplementary Data 4), it was determined that HIF-2α in BEAS-2B also plays a significant role in maintaining cell survival, consistent with our data (unpublished observations).

## Discussion

Herein, lung bronchial epithelial cells (non-malignant cells) constitutively expressing HIF-2α within the nucleus were shown to exhibit a few markers and weak features of pluripotency. Global analysis of enrichments of HIF-2α was performed to explore the diverse functions of HIF-2α under normoxia to contrast with those described for cancer cell lines 786-O and MCF-7 under hypoxia. This provided *de novo* loci enriched by HIF-2α under normoxia. Interaction with protein complexes via the N-TAD (or other domains) was found to mediate non-canonical diverse transcription functions of HIF-2α which is different from the well-studied canonical HRE enrichment via interaction of the C-TAD with P300 under hypoxia. Overall the cooperation between domains played a specific role in improving functions. Τargeting specificities under normoxia apparently extend wider range due to the diverse interactions of HIF-2α domains with other important proteins. The targeting mechanism under normoxia could also involve interactions between domains of HIF-2α with different specific co-activators. Furthermore, interactions between the enriched loci and those beyond the closest genes are conceivably likely via a chromatin looping structure^54^. The study of the hypoxia effect reported in numerous publications may have obscured non-canonical enrichments and resulted in the interpretation of the altered gene expressions as hypoxia inducible features via C-TAD activation. In fact, it may have been due to a change in abundance of HIF-2α and could be via interaction with other domain function. Hypoxia may also affect some of HIF-2α activities by epigenetic regulation apart from competition from HIF-1α, especially those involving oxygen dependent chromatin demethylase. In this study, enrichment of HIF-2α was not detected at the reported promoter core sequence of Oct-4 either under normoxia or hypoxia, in contrast to the hypoxia activated HIF-2α and expression of Oct-4 reported for (cancer) stem cells. Knockdown of HIF-2α reduced Oct-4 expression which was subject to epigenetic regulation but not hypoxia. Thus, a cell dependent unknown locus could be directly or indirectly enhanced by HIF-2α to activate Oct-4 transcription via long range interaction of chromatin. Actually, the reported enrichment levels of HIF-2α on loci within promoters of Oct-4 and other pluripotency-related genes are very low (<0.03%) in stem cells under hypoxia^40^. It is possible that *de novo* non-canonical loci which are less or un-responsive to hypoxia could be overlooked when aiming for hypoxia inducible features using an approach such as microarray or RT-PCR analysis. ChIP-Seq analyses have led to an increasing appreciation of the significance of cell and context specific enrichment of HIFs. At the same locus, protein partners co-enriched with HIFs could be context dependent. Thus, the correlation between enrichment of HIF-2α and the expression of regulated genes is not only context and cell dependent but also target dependent. It is of interest whether non-cancer cells like BEAS-2B expressing constitutive HIF-2α could assume epigenetic privilege plus environmental cues to favor pluripotency rather than malignancy. Elucidation of the regulatory mechanism of each pseudohypoxia function of constituent functional HIF-2α should be useful for resolving its role in malignancy or pluripotency in the growing number of cancer/stem cells accommodating constituent HIF-2α. Thereafter, a somatic cell which constitutively expresses HIF-2α would seem a likely candidate for developing induced pluripotent stem cells. Exploitation of the potential pluripotency of BEAS-2B cells could be a valuable model study.

ChIP-seq data is available at http://www.ncbi.nlm.nih.gov/geo/query/acc.cgi?acc=GSE81635.

## Methods

### Cell culture

The BEAS-2B cell line was obtained from ATCC and routinely cultured in RPMI1640 medium (Gibco) with high glucose containing 10% FBS (Gibco) and 2g/L sodium bicarbonate (Wako Pure Chemical Industries). The HEK293 cell line from ATCC was cultured in high glucose DMEM medium (Gibco) containing 10% FBS and 3.7g/L sodium bicarbonate.

### Western blotting

The Nuclear/Cytosol Fractionation Kit (BioVision) was used for nuclear fractionation. Extracted proteins were separated by running SDS-PAGE and transferred to polyvinylidene fluoride (PVDF) membrane. After the membrane was blocked in 5% fat-free milk in 1 × TBST and washed three times with 1 × TBST, it was immersed in 5% fat-free milk in 1 × TBST solution containing a primary antibody against HIF-2α (Novus, NB100-122; 1:200), Oct-4 (abcam, ab19857; 1 μg/ml), Nanog (ThermoFisher, PA1-097; 1:2000), Beta-tubulin (Novus, NB600-936; 1:1000), TATA binding protein (Novus, NB500-700; 1:1000), or Beta-actin (Novus, NB600-501; 1:5000), respectively at 4 °C overnight. After the membrane was washed three times with 1 × TBST, a secondary antibody against HRP-conjugated goat polyclonal anti-rabbit IgG (1:10000) or HRP-conjugated rabbit polyclonal anti-mouse IgG (1:10000) were added for 30 minutes at room temperature (RT). Finally, results were photographed by using LAS-3000 (FUJIFILM).

### Chromatin immunoprecipitation and polymerase chain reaction

Magna ChIP-Seq™ ChIP and NGS Library Preparation Kit (Millipore) were used following manufacturer’s instructions except for cell lysis buffer and nuclei lysis buffer. Five hundred and fifty microliters of 37% formaldehyde was added to a final concentration of 1% (V/V) for crosslinking of DNA and protein in BEAS-2B cells under normoxia for 10 minutes at RT. To stop this reaction, 0.125 M glycine (final concentration) was added and thoroughly mixed for 5 minutes. Ice-cold 1 × PBS containing protease inhibitor cocktail (Thermo Scientific™) was used to wash cells twice. Cells, (approximately 7.5 × 10^6^), were harvested in 1.5 ml ice-cold PBS and centrifuged at 800 × g for 5 minutes at 4 °C, followed by incubation in 1 ml cell lysis buffer (0.5% NP40, 85 mM KCl, 20 mM Tris-HCl, pH8.0) with protease inhibitors for 30 minutes on ice, and vortex-mixed briefly every five minutes. Cells were collected by centrifugation at 800 × g for 5 minutes at 4 °C, and re-suspended with 500 μl of nuclei lysis buffer (1% SDS, 10 mM EDTA, 50 mM Tris-HCl, pH8.0) containing protease inhibitors. Chromatins were sheared by using BioRuptor (Diagenode) to yield fragments mostly within 100–300 base pair range. Centrifugation at 10,000 × g was performed for 10 minutes at 4 °C after the ultrasonic shearing. The supernatant was divided into 50 μl aliquots. For immunoprecipitation, each aliquot was added to 450 μl of the dilution buffer, protease inhibitors, HIF-2α antibody (Novus, NB100-122), or p300 antibody (Novus, NB500-161), or SP1 (Millipore, 07–645) or IgG antibody (Millipore, 12–371) , together with 20 μl of protein A/G magnetic beads for subsequent incubation at 4 °C with agitation overnight. PureProteome Magnetic Stand (Millipore) was used to adsorb bead-immune complexes in each tube. These were washed sequentially by low salt-, high salt-, LiCl-immune complex wash buffer and TE buffer. Elution buffer with RNase A was added at 37 °C for 30 minutes and then proteinase K was added at 62 °C for 2 hours to elute the immune complexes from beads. The crosslinks of the immune complexes were disrupted by incubation at 95 °C for 10 minutes. DNA purification was conducted by utilizing commercial spin columns (Millipore) following the manufacturer’s instructions. Lastly, eluted DNA aliquots in TE buffer were stored at −20 °C immediately until needed. For the ChIP-PCR assay, input DNA or antibody-enriched DNA fragments were amplified with primers (Supplementary data 1 Table S1) using PCR with reagent mixture (VELOCITY DNA Polymerase, BIOLINE) according to manufacturer’s instructions. PCR products were analyzed on a 2% agarose gel. The signal strength was estimated and quantified by using ImageJ software. Results are presented as percentage input (=ChIP/input), relative fold binding, or the ratio of individual percentage input under hypoxia divided by that under normoxia.

### ChIP-sequencing and data analysis

ChIP-sequencing (ChIP-Seq) and basic bioinformatics analysis were executed by the Center for Genomic Medicine of Taiwan National Cheng Kung University. These tasks included library construction, emulsion PCR, and sequencing analysis conducted by using 5500xl SOLiD sequencer (Applied Biosystems). SOLiD Accuracy Enhancement Tool (SAET) was used to analyze raw sequence data, including enhancement of color call accuracy and mapping reads to GRCh37/hg19 by employing LifeScope™ Genomic Analysis Software. CisGenome software v2.0^55^ was used to identify binding peaks from mapped reads set at least 30 reads each window and minimum of 30 percent of total reads was limited for each single direction. Peaks were retained only when the false discovery rate (FDR) was less than 0.05 and the maximal |log2(fold change)| for all 100 bp windows within the peak was more than or equal to 5^56^. Deduction of putative HIF-2α target genes was also accomplished by performing CisGenome software v2.0. In motif analysis, MEME-ChIP and MEME were applied to identify HIF-2α binding motifs^44^,^45^. For heterodimer binding motif, dyad analysis of Regulatory Sequence Analysis Tools (RSAT) was utilized to explore potential HIF-2α partners referring to the guide to the software^57^,^58^. In addition, MAST was used for match of motifs discovered in BEAS-2B with that in previously published ChIP-seq data of 786-O under hypoxia^46^. Among the motifs, PWM Tools^47^ was used to scan the HOCOMOCO^48^ for EPAS1 motif and ARI3A motif against BEAS-2B and 786-O. In Gene ontology (GO) analysis, “The Database for Annotation, Visualization and Integrated Discovery (DAVID)” was used to explore the biological denotation of putative HIF-2α downstream genes^59^,^60^. Ingenuity Pathway Analysis^61^ was performed for analysis of pathway and “Diseases and Functions”.

### Reporter assay

All procedures followed the instructions in the Pierce™ Cypridina-Firefly Luciferase Dual Assay Kit (Thermo Scientific) manual. Briefly, pMCS-Cypridina Luc Vector inserted with or without HIF-2α enriched locus, ID 49 (ELN), 62 (IL10), 201 (PPP2R3A) or 402 (ANKRD31) together with red firefly luc control plasmid were co-transfected into BEAS-2B for 24 hours and either further exposed to normoxia or hypoxia mimicking conditions (150 μM cobalt chloride) for 4 hours. Primers are listed in Supplementary Data 1 Table S1. Backbone vector was used for background control. HIF-2α variants denoted as M2, dN, and dC were purchased from Addgene and were co-transfected with each pMCS-Cypridina Luc Vector with or without the above insert and red firefly luc control plasmid into HEK293 under normoxia as described below. Data were normalized to that of red firefly luciferase that was yielded from pTK-Red Firefly Luc Vector. Measurement of luciferase activity was conducted by using FLx800 (BioTek). M2 is a plasmid for expression of full length of active HIF-2α in which the amino acid sequences was mutated at position 405 (P- > A), 531 (P- > A), and at 847 (N- > A). dN is a plasmid for expression of the N-TAD (450–572) deleted variant of HIF-2α and is mutated at 405 (P- > A). dC is a plasmid for expression of the C-TAD (820–870) deleted variant of HIF-2α and is mutated at position 405 (P- > A) and 531 (P- > A)^50^.

### RT-PCR and Knockdown

RNA was extracted by using REzol^TM^ C&T following the manufacturer’s instruction. Next, High-Capacity cDNA Reverse Transcription Kit (Applied Biosystems™) was used to reverse transcribe RNA to cDNA. Master Mix Kit 2.0X (Topbio PCR MIX) was used to conduct PCR and signal intensity was quantified by using ImageJ software. Data was normalized to beta-actin. For Knockdown of HIF-2α, BEAS-2B cells were transfected with shHIF-2α (TRCN000003806) or shLuc (TRCN0000072245) by using lipofectamine 2000 reagent (Invitrogen) according to the manufacturer’s protocol. RNA was extracted after one day of transfection. Primers are listed in Supplementary Data 1 Table S1.

### Sphere culture

First, BEAS-2B cells were cultured as a monolayer as above. Then, cells were harvested and around 20,000 cells/well were seeded into Ultra-low Attachment 6-well plates (Corning , Corning, NY) containing DMEM/F12 (Gibco) with 20% KnockOut™ Serum Replacement (Gibco), 1X Non-essential amino acids (Biological Industries), 1 mM L-alanyl-L-glutamine (Biological Industries), 100 μM 2-mercaptoethanol (Sigma), and 10 ng/ml recombinant human fibroblast growth factor-basic (PEPROTECH). Two hundred and fifty microliters of fresh medium was added every three days. Spheres were photographed on day 10.

### Statistical analysis

Data are shown as mean ± the standard deviation. T-test was used to determine statistical significance (*P* < 0.05) manipulated using Excel.

## Additional Information

**How to cite this article**: Lee, M.-C. *et al.* Genome-wide analysis of HIF-2α chromatin binding sites under normoxia in human bronchial epithelial cells (BEAS-2B) suggests its diverse functions. *Sci. Rep.* **6**, 29311; doi: 10.1038/srep29311 (2016).

## Supplementary Material

Supplementary Dataset 1

Supplementary Dataset 2

Supplementary Dataset 3

Supplementary Dataset 4

## Figures and Tables

**Table 1:** Top 20 of HIF-2α enriched sites and physically annotated gene loci.

Rank^1^ (ID)	Chromosome	Start	End	Closest target gene	RS No.
1	chr17	1505243	1505491	SLC43A2	NM_152346
2	chr5	108710644	108710842	PJA2	NM_014819
3	chr9	80468868	80469140	GNAQ	NM_002072
4	chr4	104324881	104325132	TACR3	NM_001059
5	chr14	68913870	68914070	RAD51L1	NM_133510
6	chr2	207427980	207428203	ADAM23	NM_003812
7	chr9	109689828	109690038	ZNF462	NM_021224
8	chr5	106573204	106573412	EFNA5	NM_001962
9	chr3	33219585	33219788	SUSD5	NM_015551
10	chr4	30464070	30464272	PCDH7	NM_032456
11	chr21	26735272	26735471	NCRNA00158	NR_024027
12	chr10	64625240	64625442	EGR2	NM_001136178
13	chr19	10530240	10530440	PDE4A	NM_001111307
14	chr15	55805854	55806054	DYX1C1	NM_130810
15	chr6	9578174	9578417	TFAP2A	NM_001032280
16	chr15	79587779	79588012	ANKRD34C	NM_001146341
17	chrUn	139808	140140	–2	–2
18	chr15	64934178	64934376	ZNF609	NM_015042
19	chr10	114444062	114444260	VTI1A	NM_145206
20	chr2	217496604	217496818	IGFBP2	NM_000597

^1^Binding peaks ordered by fold enrichment from high to low.

^2^No corresponding gene.
