# Supplementary material for: Genome-wide analysis of HIF-2α chromatin binding sites under normoxia in human bronchial epithelial cells (BEAS-2B) suggests its diverse functions
Source: Sci Rep. 2016 Jul 4;6:29311. doi: 10.1038/srep29311 (PMC4931692; doi:10.1038/srep29311)

**Genome-wide analysis of HIF-2α chromatin binding sites under normoxia in human bronchial epithelial cells (BEAS-2B) suggests its diverse functions**

Meng-Chang Lee^1^, Hsin-Ju Huang^2^, Tzu-Hao Chang^3^, Hsieh-Chou Huang^2,6^, Shen-Yuan Hsieh^1^, Yi-Siou Chen^4^, Wei-Yuan Chou^4^, Chiao-Hsi Chiang^7^, Ching-Huang Lai^5^ and Chia-Yang Shiau*^,1,4,5^

**Table S1** Primer sequences

| Binding ID (Annotated gene) or  target | Forward | Reverse |
| --- | --- | --- |
| 49 (ELN) | GTACACCACCGGAAAGCAA | AGTCGATGCTGGAGCTGAAT |
| 62 (IL10) | GGGTAGACAAGGGAGAAGCC | GCTGCTAAACTGAAAGACCACC |
| 144 (JMJD5) | GTGCAAAGATCCCAAGGCAT | GCATTACATCAAAACCCAAACCC |
| 151 (PDXK) | GGATATCTGTCACTGGGCATC | GACAGGGAACCTTCCAGAGA |
| 164 (SCG2) | TCCATCTCTAGCCCAGACTTCT | CATGTTGGATTGCGCTGACA |
| 201 (PPP2R3A) | TCCTGCTTCTTCACTCCGTAC | CAGGGTACATGACTTGGGCT |
| 307 (SUDS3) | TCCAGTTTTACCCCTTTCTGACA | CTCACCACATCCAACTGCCT |
| 402 (ANKRD31) | AAGGTTAGAGAGCATTGGGAGG | TCTGGCCCTAACATAACCTTTCA |
| 492 (SP3) | AATGCTTTCTACATGGGTTGGC | AGAGTTGAGCCAAAATCTGCTG |
| 793 (CBY3) | CCCTGAAGACGGACCAAGTG | GGAGGACGGTGTATGGGGT |
| *Negative control | ACCATGGATGATGATATCGCC | GCCTTGCACATGCCGG |
| 49 (ELN) for reporter assay | GGAATTCCCAGCGTCCTCTGAGAGTCTGTTAC | CGGGATCCCGGGGTCAGAGCCTGCA |
| 62 (IL10) for reporter assay | GGAATTCCATCAAAGTGTGGCCTCA | CGGGATCCCGAAACCACAAACACCG |
| 201 (PPP2R3A) for reporter assay | GGAATTCCACAATTTTATTCTGTTGTGAATGT | CGGGATCCCGAACAGCTTTCCTGTAT |
| 402 (ANKRD31) for reporter assay | GGAATTCCTTATCTTTAAAATACAGTCATAGTC | CGGGATCCCGCTAGGCCCAGTATTCA |
| Beta-actin for RT-PCR | GCCGCCAGCTCACCAT | CACGATGGAGGGGAAGACG |
| **Oct-4 for RT-PCR | GAGAACCGAGTGAGAGGCAACC | CATAGTCGCTGCTTGATCGCTTG |
| ***HIF-2α for RT-PCR | GGACTTACACAGGTGGAGCTA | TCTCACGAATCTCCTCATGGT |

*Negative control primers are of beta-actin of human, referring to Schodel et al (2011).

** Primers are of Oct-4 of human, referring to Pietras et al (2009).

*** Primers of HIF-2α of human were obtained by using PrimerBank (https://pga.mgh.harvard.edu/primerbank/).

**Table S2** ChIP-Seq reads count and mapping summary.

| Sample | Total Reads  (75, 35 reads no.) | Mapped Reads*  (75, 35 reads no.) | Mapping% | Mapping QV  (Avg;Median) | Base QV**  (Avg;Median) |
| --- | --- | --- | --- | --- | --- |
| HIF2α | 14,967,595  (8910617, 6056978) | 10,313,454  (6936551, 3376903) | 68.91 | 56.48 ; 72.2 | 36.27 ; 41 |
| IgG | 12,978,027  (8543944, 4434083) | 10,469,717  (7560009, 2909708) | 80.67 | 55.49 ; 69.6 | 36.18 ; 41 |

*Mapped Reads: reads mapping to the Genome Reference Consortium GRCh37 (hg19).

** Base QV (quality value): quality value of each base after sequencing.

When the base QV is equal to 40, a possible error rate is 1/10,000 bases. Forty one means an error probability lower than 1/10,000 bases.

**Table S3 A**

A list of common genes annotated to HIF-2α enriched loci in BEAS-2B and MCF-7.

| Item | Gene | Accession number | Item | Gene | Accession number |
| --- | --- | --- | --- | --- | --- |
| 1 | SLC2A3 | NM_006931 | 25 | DDIT4L | NM_145244 |
| 2 | EGFR | NM_201283 | 26 | ROBO1 | NM_002941 |
| 3 | PXDN | NM_012293 | 27 | SOX9 | NM_000346 |
| 4 | IRX1 | NM_024337 | 28 | NCOR2 | NM_006312 |
| 5 | SCARB1 | NM_005505 | 29 | SWAP70 | NM_015055 |
| 6 | ARRDC3 | NM_020801 | 30 | HS6ST3 | NM_153456 |
| 7 | CITED2 | NM_006079 | 31 | NR2F2 | NM_021005 |
| 8 | SLC28A1 | NM_201651 | 32 | UBE2E3 | NM_182678 |
| 9 | PKM2 | NM_002654 | 33 | CXCR7 | NM_020311 |
| 10 | PARN | NM_002582 | 34 | ENO1 | NM_001428 |
| 11 | ATP9A | NM_006045 | 35 | TLE4 | NM_007005 |
| 12 | PSMD6 | NM_014814 | 36 | ACTL7B | NM_006686 |
| 13 | STC2 | NM_003714 | 37 | ARHGAP26 | NM_015071 |
| 14 | HEY1 | NM_012258 | 38 | C9orf50 | NM_199350 |
| 15 | PRDM1 | NM_001198 | 39 | TRIOBP | NM_001039141 |
| 16 | SUMF1 | NM_182760 | 40 | C3orf32 | NM_015931 |
| 17 | C14orf43 | NM_194278 | 41 | KRT7 | NM_005556 |
| 18 | GBE1 | NM_000158 | 42 | WASL | NM_003941 |
| 19 | PAG1 | NM_018440 | 43 | FAM102A | NM_001035254 |
| 20 | KCNMA1 | NM_002247 |  |  |  |
| 21 | IRF2BP2 | NM_001077397 |  |  |  |
| 22 | DYRK1A | NM_001396 |  |  |  |
| 23 | CXXC5 | NM_016463 |  |  |  |
| 24 | QKI | NM_206855 |  |  |  |

**Table S3 B**

A list of common genes annotated to HIF-2α enriched loci in BEAS-2B and 786-O.

| Item | Gene | Accession No. | Item | Gene | Accession No. | Item | Gene | Accession No. | Item | Gene | Accession No. |
| --- | --- | --- | --- | --- | --- | --- | --- | --- | --- | --- | --- |
| 1 | MYEOV | NM_138768 | 26 | PTGES | NM_004878 | 51 | TGM2 | NM_198951 | 76 | FLJ43860 | NM_207414 |
| 2 | TLR4 | NM_138554 | 27 | GABRR2 | NM_002043 | 52 | PGPEP1 | NM_017712 | 77 | SH3BP4 | NM_014521 |
| 3 | SLC28A3 | NM_022127 | 28 | CCDC91 | NM_018318 | 53 | ATP9A | NM_006045 | 78 | UBE2D2 | NM_003339 |
| 4 | KCNMA1 | NM_002247 | 29 | CUEDC1 | NM_017949 | 54 | ITPR1 | NM_002222 | 79 | CRISPLD2 | NM_031476 |
| 5 | PHACTR1 | NM_030948 | 30 | EGFR | NM_005228 | 55 | CITED2 | NM_006079 |  |  |  |
| 6 | RASSF4 | NM_032023 | 31 | GDF6 | NM_001001557 | 56 | BTBD16 | NM_144587 |  |  |  |
| 7 | SLC28A1 | NM_004213 | 32 | ADAMTS16 | NM_139056 | 57 | MICALCL | NM_032867 |  |  |  |
| 8 | ZNF365 | NM_199452 | 33 | GTF3C1 | NM_001520 | 58 | PXDN | NM_012293 |  |  |  |
| 9 | BICD1 | NM_001003398 | 34 | RB1CC1 | NM_014781 | 59 | FUT9 | NM_006581 |  |  |  |
| 10 | EFNA5 | NM_001962 | 35 | CUL4A | NM_001008895 | 60 | MAEA | NM_001017405 |  |  |  |
| 11 | TNFSF10 | NM_003810 | 36 | MKI67IP | NM_032390 | 61 | RAMP3 | NM_005856 |  |  |  |
| 12 | PDE10A | NM_006661 | 37 | DNAH5 | NM_001369 | 62 | SLC39A11 | NM_139177 |  |  |  |
| 13 | IRF2BP2 | NM_001077397 | 38 | PDXK | NM_003681 | 63 | UBE2E2 | NM_152653 |  |  |  |
| 14 | NR6A1 | NM_001489 | 39 | COL18A1 | NM_130445 | 64 | COL1A2 | NM_000089 |  |  |  |
| 15 | CAV2 | NM_001233 | 40 | DIP2C | NM_014974 | 65 | FRMD4A | NM_018027 |  |  |  |
| 16 | SULF1 | NM_015170 | 41 | STAC | NM_003149 | 66 | ARRDC3 | NM_020801 |  |  |  |
| 17 | SLC38A1 | NM_030674 | 42 | LARS2 | NM_015340 | 67 | DOK5 | NM_018431 |  |  |  |
| 18 | STC2 | NM_003714 | 43 | UBE2O | NM_022066 | 68 | FOSL2 | NM_005253 |  |  |  |
| 19 | DAB2IP | NM_032552 | 44 | SLC2A3 | NM_006931 | 69 | PAG1 | NM_018440 |  |  |  |
| 20 | AHDC1 | NM_001029882 | 45 | NRIP1 | NM_003489 | 70 | PRKAG2 | NM_001040633 |  |  |  |
| 21 | ALCAM | NM_001627 | 46 | LATS2 | NM_014572 | 71 | TRIP13 | NM_004237 |  |  |  |
| 22 | DAPK1 | NM_004938 | 47 | GNA13 | NM_006572 | 72 | ERGIC1 | NM_001031711 |  |  |  |
| 23 | KIAA1026 | NM_001017999 | 48 | SIPA1L3 | NM_015073 | 73 | CACNA2D3 | NM_018398 |  |  |  |
| 24 | SDHA | NM_004168 | 49 | DAD1 | NM_001344 | 74 | PGRMC2 | NM_006320 |  |  |  |
| 25 | FZD8 | NM_031866 | 50 | ADAM12 | NM_021641 | 75 | SEMA6A | NM_020796 |  |  |  |

**Figure S1** Treatment of TSA enhances expression level of Oct-4 in BEAS-2B.

**A**

Control

TSA

Relative intensity: 1.0 1.25


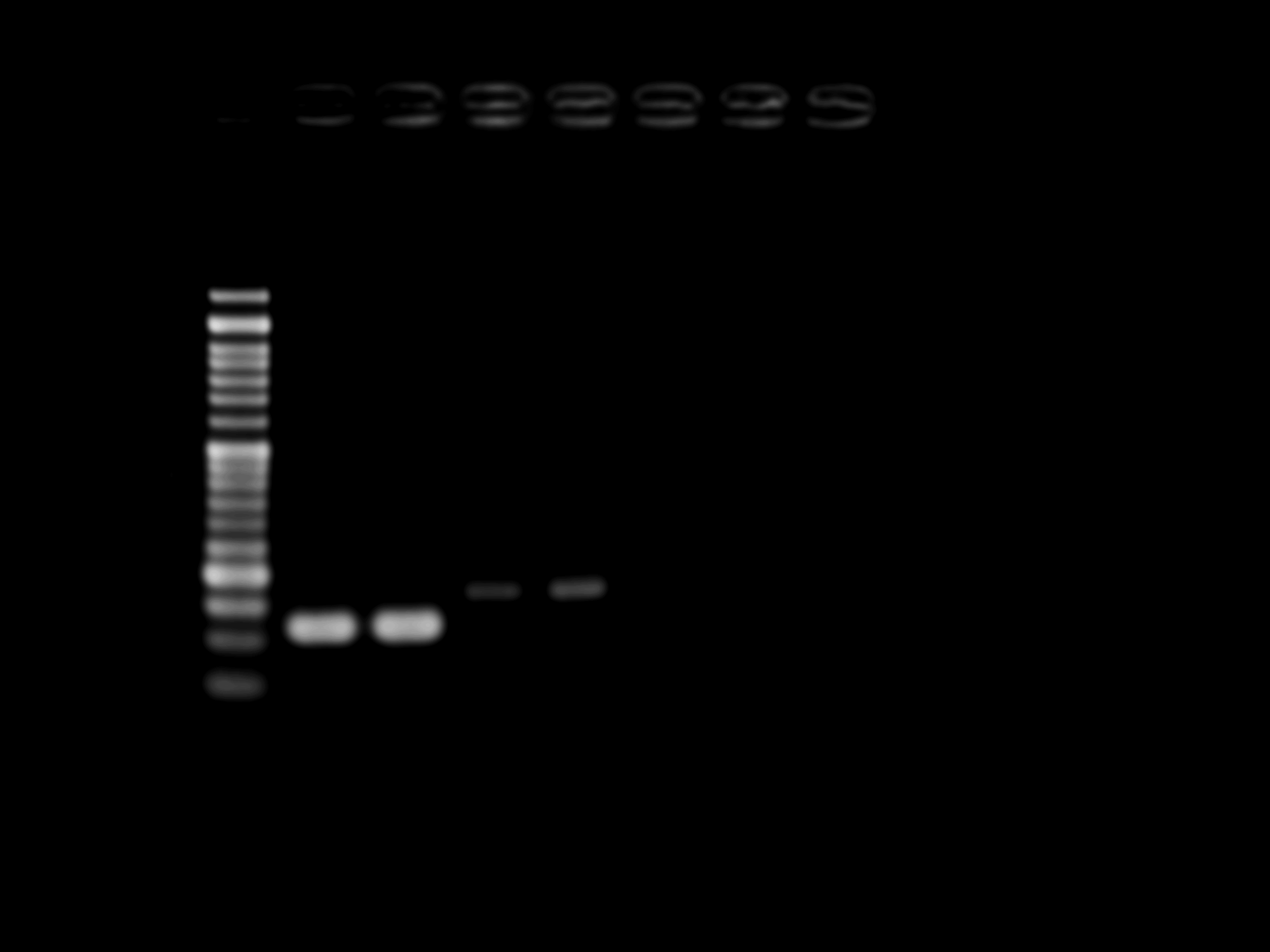


Oct-4


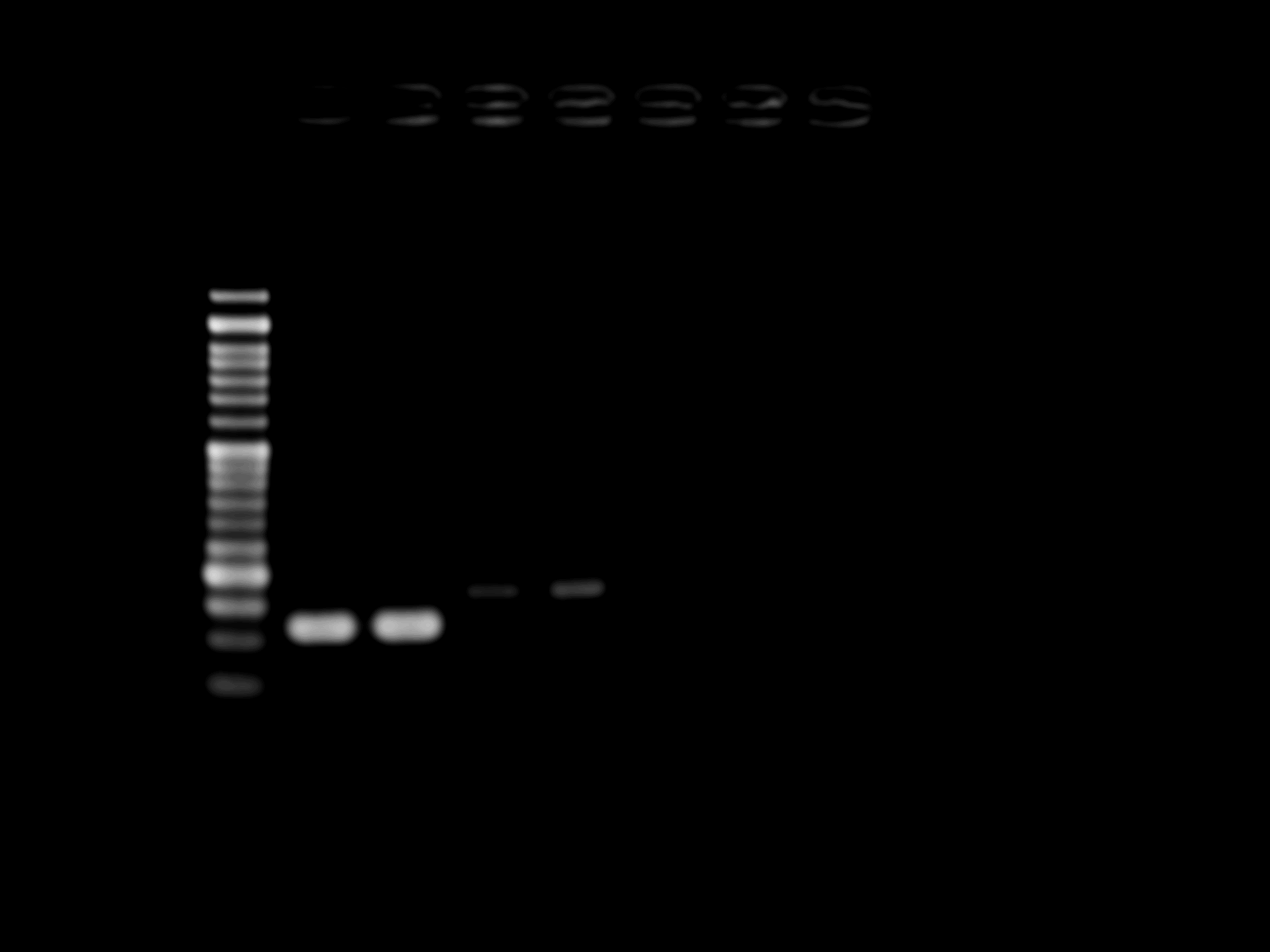


Beta-actin

**B**

Control

TSA

Relative intensity: 1.0 1.31


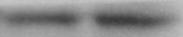


Oct4


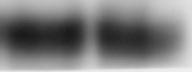


Beta-tubulin

(A) RT-PCR and (B) Western blotting assay of expression of Oct4. BEAS-2B cultured under normoxia was treated 1ng/ml TSA for 2 days while treatment of DMSO (solvent of TSA) as a control. Data was normalized to Beta-actin or Beta-tubulin.

**Figure S2** Motif analysis using MAST.

**A** Results of scanning motif 1 of BEAS-2B against 786-O (referring to closest genes).


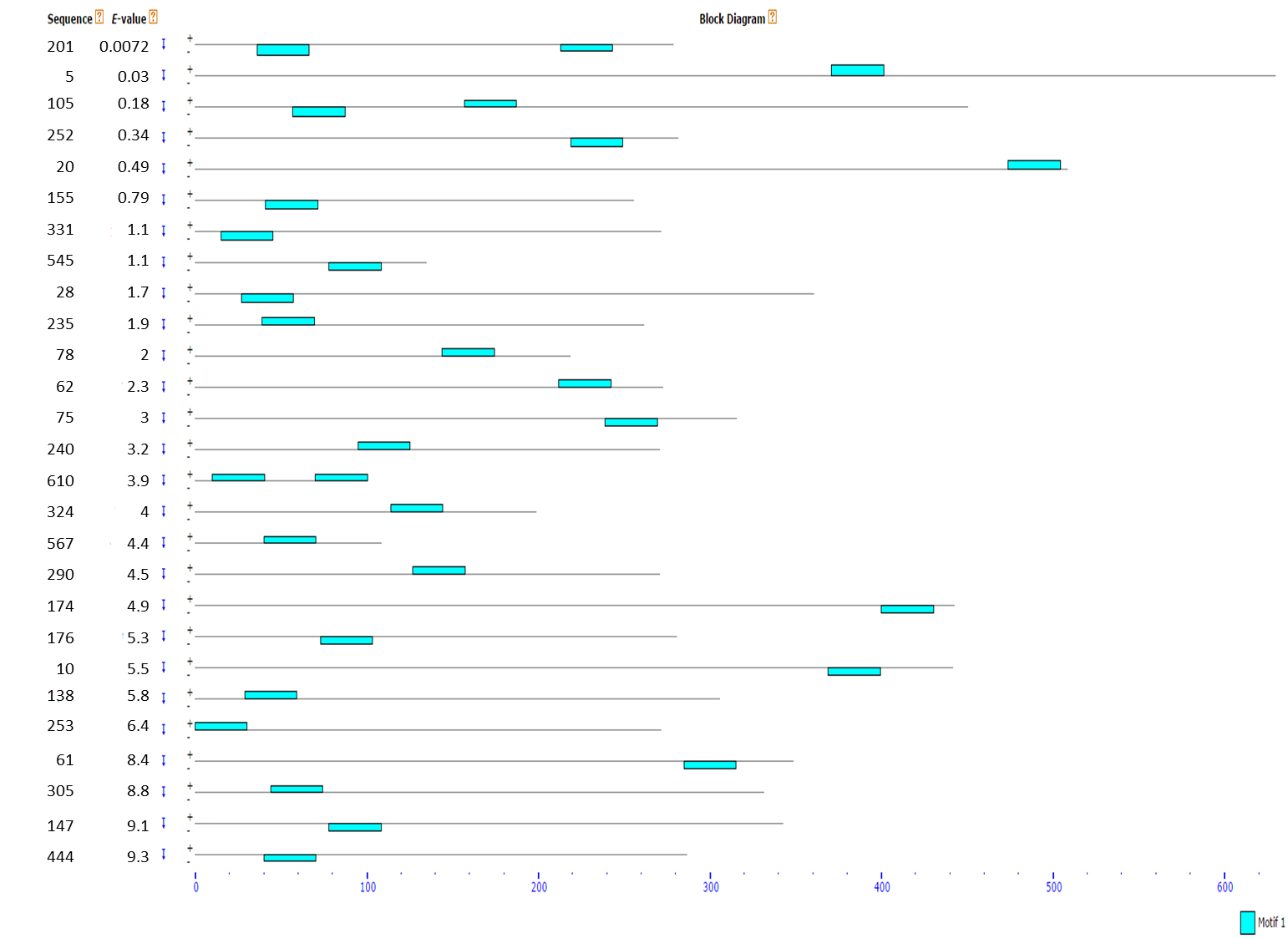


**Figure S2**

**B** Results of scanning motif 2 of BEAS-2B against 786-O (referring to closest genes).


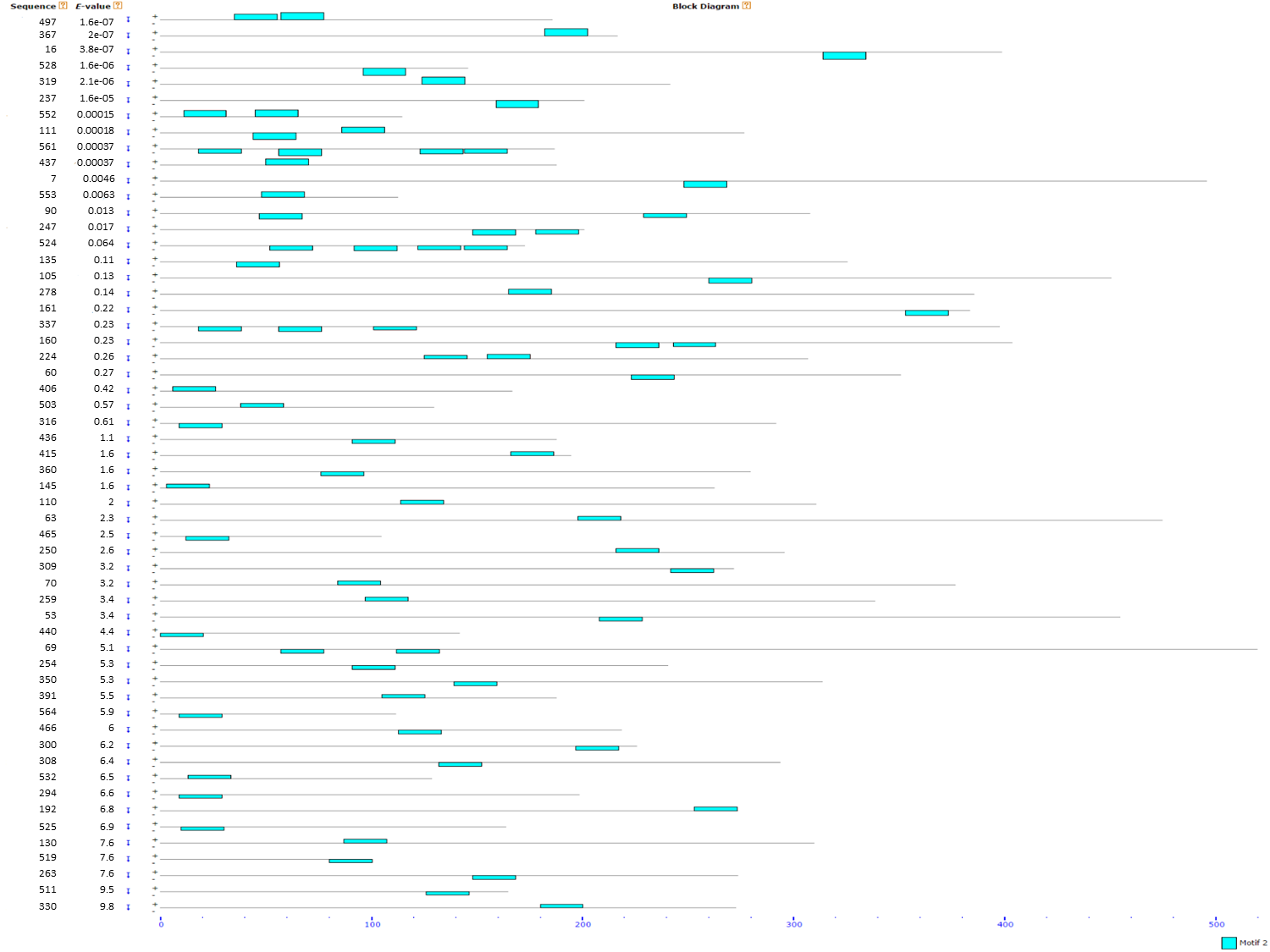


**Figure S2**

**C** Results of scanning motif 3 of BEAS-2B against 786-O (referring to closest genes).


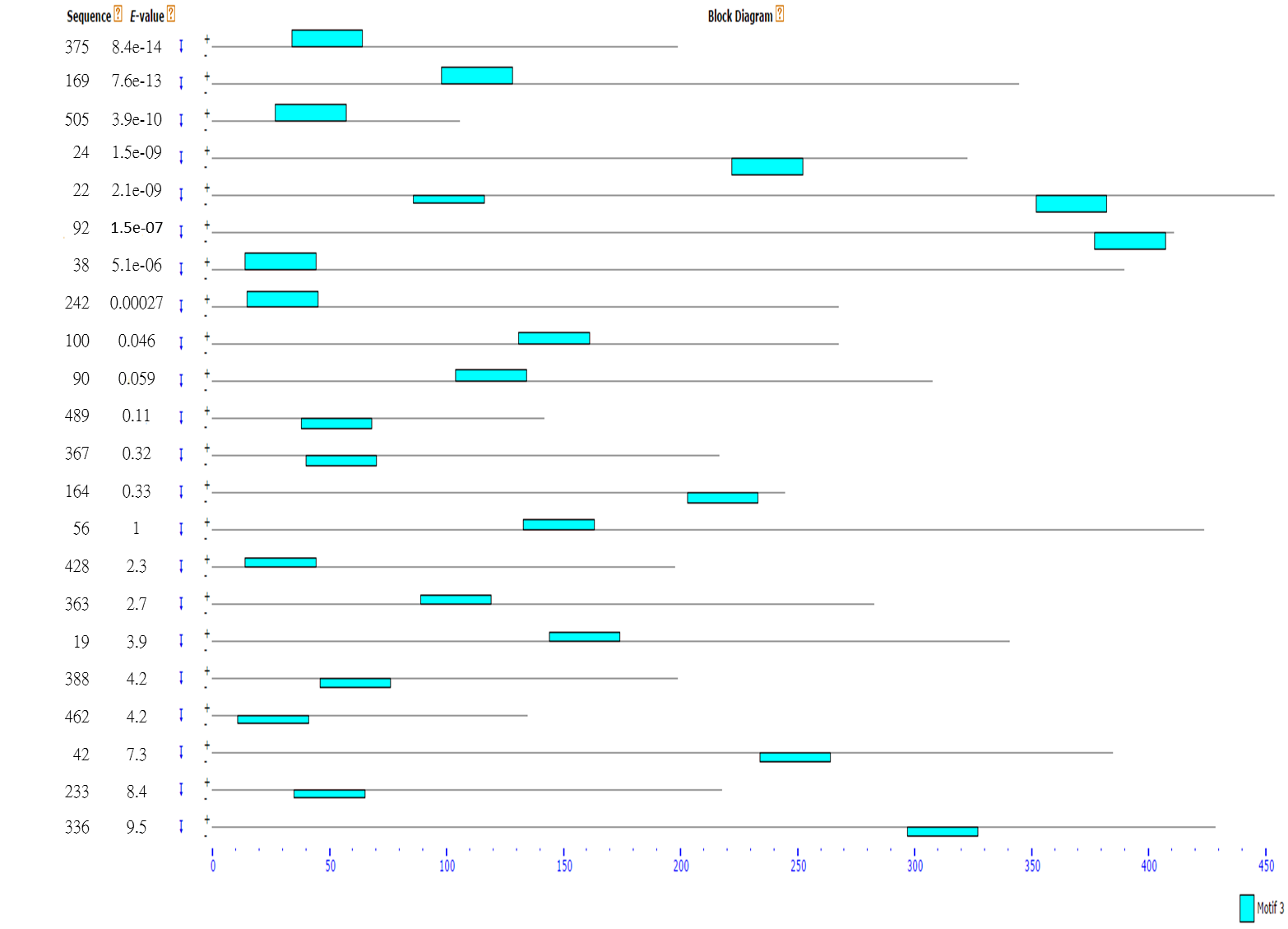

Supplement: Supplementary Dataset 1 [file srep29311-s1.doc]
